# Selective venous sampling in primary hyperparathyroidism: Is it worth doing?

**DOI:** 10.3906/sag-2108-151

**Published:** 2021-10-17

**Authors:** Selda GÜCEK HACİYANLI, Nihan ACAR, Ömür BALLI, Nezahat ERDOĞAN, Mehmet HACİYANLI

**Affiliations:** 1 Department of General Surgery, İzmir Katip Çelebi University Atatürk Training and Research Hospital, İzmir, Turkey; 2 Department of Radiology, İzmir Katip Çelebi University Atatürk Training and Research Hospital, İzmir, Turkey

**Keywords:** Persistent, primary hyperparathyroidism, venous sampling, 4D-CT, 4D-MRI, parathyroidectomy

## Abstract

**Background/aim:**

Accurate preoperative localization of the culprit gland is the key point for the surgical treatment of primary hyperparathyroidism. Conventional imaging techniques (ultrasound and Tc99m sestamibi scintigraphy) are usually adequate for preoperative localization. However, in some patient groups, additional imaging modalities may be required since noninvasive techniques may fail. In this study, we aimed to evaluate the diagnostic value of selective parathyroid venous sampling in patients with unclear noninvasive localization tests.

**Materials and methods:**

Among 513 cases who underwent parathyroidectomy due to primary hyperparathyroidism, twelve cases (2.3%) were undergone selective parathyroid venous sampling and were included in the study. Age, sex, presenting symptom, presence of a genetic disease, medical and surgical history, serum calcium (Ca)-parathormone (PTH) levels (preoperative, intraoperative, and postoperative), imaging reports (US, SM, and SVS), surgery reports, pathology reports, and complications were retrospectively reviewed.

**Results:**

Seven cases (58.3%) had persistent primary hyperparathyroidism and one patient (8.3%) had past surgical history of total thyroidectomy. The remaining four patients (33.3%) had no previous neck surgery.

The sensitivity of selective venous sampling was 75%. According to the medical history, accurate localization was achieved in 85.7% of persistent cases and 60% of primary cases. Eight cases (66.6%) underwent unilateral neck exploration and four cases (33.3%) underwent four gland exploration. A single adenoma was detected in ten cases (90.9%) while one patient (9.1%) had double adenoma.

**Conclusion:**

Selective venous sampling is a prominent technique with high accuracy in persistent cases when the conventional imaging methods are negative or equivocal. Although the cost and invasiveness are the most common confusing facts about selective venous sampling, its benefits such as improving the surgical success and saving the patients from repeated operations may surpass them.

## 1. Introduction

Primary hyperparathyroidism (PHPT), which mostly occurs due to a solitary parathyroid adenoma (PA) (80%–87%), is a common endocrine disorder [1]. Its curative treatment is the excision of the hyperfunctioning gland(s). There are several surgical options varying from minimally invasive parathyroidectomy to four-gland exploration. Less invasive techniques, which are also introduced as focused parathyroid surgery, consist of minimally invasive parathyroidectomy, endoscopic parathyroidectomy, and unilateral neck exploration. These approaches offer less pain, shorter hospital stay, faster recovery, and fewer complications when compared to conventional four-gland exploration [2]. Since the most common cause is single adenoma, accurate preoperative localization of the culprit gland is the key point to achieving a focused approach.

First line imaging methods such as ultrasound (US) and Tc99m sestamibi scintigraphy (SM) are usually adequate for preoperative localization. In cases which these modalities are negative or discordant, second line imagings including four dimensional (4D) computed tomography (CT) and magnetic resonance imaging (MRI) are preferred. All these noninvasive imaging techniques may fail in some patient groups, especially in persistent and recurrent cases. Selective venous sampling (SVS) is an advanced invasive technique that is based on the consecutive blood sampling from cervical veins and parathormone (PTH) measurement in these samples and aid to localize the hyperfunctioning gland [3] in persistant/recurrent cases and even in primary cases [4].

In this study, we aimed to evaluate the diagnostic value of SVS in patients with the unclear noninvasive localization tests in primary and persistant/recurrent PHPT.

## 2. Materials and methods

The medical records of 513 cases who underwent parathyroidectomy due to PHPT in our institution between 2013 and 2020were reviewed retrospectively. In our Institution, neck ultrasonography and Technetium 99m sestamibi schintigraphy are the first-line imaging series for all PHPT patients. If they fail to identify the presumable adenoma, we prefer to use the second line noninvasive imaging modalities i.e., 4D-CT, 4D-MRI. In suitable suspect lesions, measurement of PTH level in US-guided aspirate is also used.

4D-CT technique for the localization of abnormal gland in PHPT preoperatively was first described by Rodgers et al in 2006 [5] and has been used in our center since 2009. Dynamic MRI and then 4D-MRI for PHPT have been successfully used in our hospital after the report of Grayev et al. [6] in 2012, particularly for the young patients to avoid the high radiation exposure of 4D-CT.

A positive first and second line localization test means enlarged parathyroid gland with typical features (i.e., separate hypoechoic mass with polar vascularity around the thyroid gland on US, clear sestamibi accumulation on late images or early contrast enhancement, and early contrast washout on CT/MR). Equivocal test means that there is a suspicious lesion, which exibits those features partially.

If all those studies fail to clearly localize the gland in persistent/recurrent cases, or if the patient needs to clarify his/her mind to decide on surgery, we ask for the SVS from the Interventional Radiology Department.

Twelve cases were found to undergo SVS. Following parameters were recorded and assessed: Age, sex, presenting symptom, presence of a genetic disease, medical and surgical history, serum calcium (Ca)-parathormone (PTH)levels (preoperative, intraoperative, and postoperative), imaging reports (US, SM, and SVS), surgery reports, pathology reports, and complications.

Written informed consent was obtained from each patient for the publication. The study protocol was approved by the institutional review board (12.05.2020 number: 664) and was conducted in accordance with the principles of the Declaration of Helsinki.

### 2.1. Selective venous sampling

**Figure 1:**
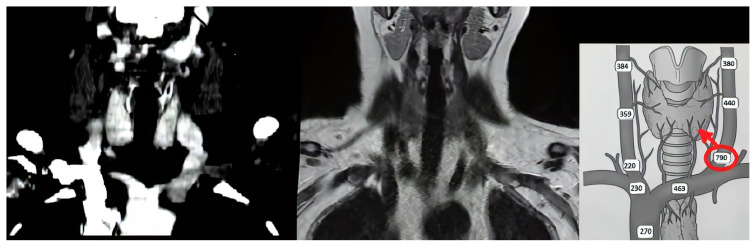
Comparative display of CT, MRI images, and SVS diagram of patient #10.

The procedure was performed under sedation anesthesia by an experienced interventional radiologist. Following the angiographic catheterization from femoral vein, blood samples were collected consecutively from superior vena cava, right-left brachiocephalic veins, right-left jugular veins, and thyroid veins in order to measure PTH levels. Results were recorded in diagrams (Figure 1). A ≥2-fold increased PTH level compared to the PTH level in blood sample from a peripheral vein was accepted as positive [7]. This was interpreted by dividing the neck into four quadrants for localizing the hyperfunctioning gland.

### 2.2. Surgical strategy

In persistent and recurrent cases, at least one precise or two suspicious but concordant imaging was required as necessary criteria for operation in our center [8]. However, there were two exceptions: patient #8 had a thyroid disease requiring bilateral thyroidectomy, so the operation was undertaken, and patient #7 had overt hypercalcemia and a strongly suspicious lesion on repeat US. Patient #6 had a very small (6 mm) presumable parathyroid gland on 4DCT and discordantly high serum calcium level (12.6 mg/dL) with that gland, so she was operated too. All patients were operated on by a single surgeon (M.H.) experienced in endocrine surgery. Intermittent intraoperative neuromonitoring (IONM) was used routinely. The image-guided unilateral focused approach or limited exploration was preferred in persistent/recurrent cases.

## 3. Results

Demographics, clinical features, and imaging findings of the patients are given in Table.

The majority of the patients (n:11, 91.6%) were female, and the mean age was 56.3years (range: 45–68). Seven cases (58.3%) had persistent PHPT, and one patient (8.3%) (Patient #7) had a past surgical history of total thyroidectomy. Remaining four patients (33.3%) had no previous neck surgery (Patients #2, #5, #9 and #11).

Four patients (33.3%) had suspicious findings in US, while aspirate PTH study was negative in them. Five patients (41.6%) were reported to have suspicious findings in SM. Eight patients (66.6%) underwent 4D-CT, and five patients (41.6%) underwent 4D-MRI as second line imaging (Patient #10 had both). According to second-line imaging results, only four of the patients (33.3%) had suspicious positive findings that might belong to a hyperfunctioning parathyroid gland.

All cases underwent SVS, and it was able to localize the correct quadrant in eight patients (66.6%). Although lateralisation was not obtained in Patient #2, significantly elevated PTH level was detected bilaterally in the level of inferior glands which was later explained with double adenoma (positive test). Patient #4 underwent SVS although there was a correlation between her SM and MRI, since she had a history of two previous unsuccessful parathyroid surgeries. Eight cases (66.6%) underwent unilateral neck exploration, and four cases (33.3%) underwent four gland exploration. A single adenoma was detected in ten cases (90.9%), while one patient (9.1%) had double adenoma. The mean diameter of 12 lesions was 1.4 ± 0.41 cm. In patient #12, although SVS indicated a positive increase in PTH in the left lower quadrant, no adenoma was found during the unilateral exploration. Therefore, the inferior thyroid artery was ligated in this case. Adequate intraoperative PTH decrease was obtained in all cases according to the Miami criterion [9].

**Figure 2:**
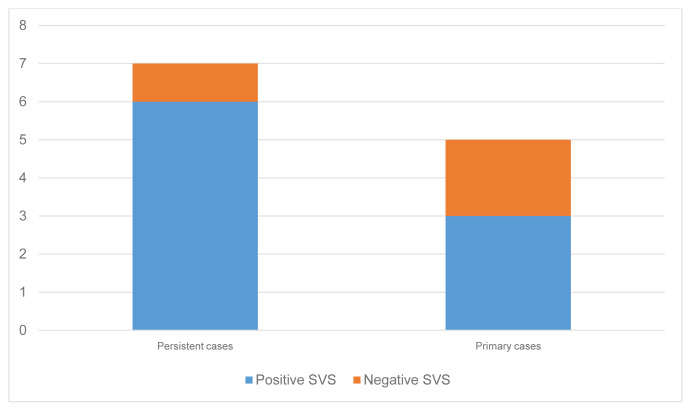
Distribution of thesuccess of SVS accordingtoclinicalfeatures of the cases.

Overall, sensitivity of SVS was 75% (9/12 patients). According to the medical history, accurate localization was achieved in 85.7% of persistent cases and 60% of primary cases (Figure 2).

None of the patients developed any complications related with SVS or surgery. Normocalcemia was maintained in all cases during the mean 58 months (range: 12–102 months) of follow-up.

## 4. Discussion

Selective venous sampling may seem a bit invasive procedure for PHPT as a preoperative diagnostic modality. However, the fact that it can become crucial in some patient groups is incontrovertible. In the literature, these special patient groups were defined as recurrent or persistent cases in which imaging study results are usually negative or discordant [7]. According to our results and observation, we can expand the indications for SVS by including the primary cases of advanced age who have comorbidities carrying high risk for surgery, by considering the benefit-loss balance (33.3% of our series consisted of primary cases with these special features).

Since 85% of PHPT cases are due to single gland disease, focused approaches are usually sufficient for the treatment instead four-gland exploration. For this reason, localization of the lesion is the most important factor. Although noninvasive imaging methods usually achieve the localization, there are also instances where they remain inadequate. Factors such as the experience of the radiologist, body mass index (BMI), and gland size were found to have an effect on the failure of the non-invasive imaging modalities[1,10,11]. None of these factors were found to contribute to the failure in our series; however, 66.6% of the cases had at least one previous neck surgery due to parathyroid and/or thyroid disease.

In cases which first and second-line imaging modalities display negative or discordant results, SVS was introduced as a third-line imaging method. Its sensitivity was reported as 77%–94.7% [7,12–14]. There are some technical factors affecting the success of SVS. In a study by Ikuno et al., sampling from all thyroid veins besides the jugular veins was shown to improve the sensitivity of SVS [14]. Nevertheless, this distal sampling is both time-consuming and technically more difficult. In our series, due to technical resources, the most distal point that the sampling could be performed was the junctions where thyroid veins drained into the jugular veins. Therefore, our sensitivity of 75% was slightly lower than the previous studies. Nevertheless, when the cases were evaluated in subgroups, sensitivity of 83.3% in persistent cases was concordant with the literature.

Previous neck surgery has been shown as a responsible for the failure in SVS because of detorioration in vascular drainage due to ligations [15,16]. Among the three patients from our series in which SVS was negative, two had previous neck surgery. However, it is hard to interpret this result with such a small number of patients.

The major disadvantages of SVS are that it is expensive, requires experience and involves radiation exposure. Besides, some complications such as groin hematomas, vascular injury and venous thrombosis can be encountered after SVS [4,17]. None of our patients developed any complications related with SVS.

In their meta-analysis, Ibraheem et al. emphasized that although SVS had higher sensitivity than noninvasive imaging modalities in reoperative parathyroid surgery, its place in routine utilization was not feasible due to its invasiveness [18]. Accuracy of SVS 75% in patients without previous neck surgery. Therefore, SVS may be a savior imaging test when conventional imagings are negative in primary cases, in order to achieve a focused approach and to avoid the risk of persistency.

In 2015, Ginsburg et al. showed that the parathyroid SVS combined with 4D-CT improves sensitivity and accuracy of identification of parathyroid adenoma in 28 patients with recurrent or persistent PHPT with negative MIBI and US scans [19]. They found that comparing 4D CT alone with 4D CT plus SVS, the sensitivity increased from 50% to 95%, and accuracy increased from 55% to 91%. Since then, very limited report has been published in the literature. Our study is unique in that aspect.

Seehofer et al. proposed that two compatible preoperative localization studies should be valid before reoperative parathyroid surgery [8]. We also share the same criteria in order to pursue a focused approach. However, surgeon experience is also another criteria that may allow to perform a focused parathyroidectomy. Therefore, 85.7% of the patients who underwent unilateral exploration had only one positive imaging in our series which was mostly provided by a successful SVS.

Its retrospective design and small sample size are the limitations of this study. Multi-center studies should be conducted in order to obtain larger sample size, since SVS is not a frequently used technique even in experienced centers.

Selective venous sampling combined with 4D-CT/MRI is a prominent technique with high accuracy in persistent cases when the conventional imaging methods are negative or equivocal. Its place in primary cases should also be evaluated in further studies. Although the cost and invasiveness are the most common confusing facts about SVS, its benefits such as improving surgical success and saving the patients from repeated operations may surpass them.

## Figures and Tables

**Table:** Clinical features and imagings of the patients.

Patientno	Age	Sex	US	SM	CT	MRI	SVS	Surgicalprocedure	Localizationof the culpritgland
#1	50	F	Neg	Neg	-	Neg	+	UE	RS
#2	68	F	Neg	LI equ	Neg	-	+	FGE	Bilat. I
#3	48	F	Neg	Neg	-	Neg	+	UE	LS
#4	67	F	Bilat. I	LI	-	L equ	+	UE	LI
#5	64	F	Neg	Neg	LS	-	Neg	UE	LI
#6	45	F	Neg	Neg	Neg	-	+	FGE	LS
#7	49	F	LI equ	Neg	-	Neg	Neg	UE	LI
#8	59	F	Neg	RI equ	Neg	-	Neg	FGE+TT	RI
#9	62	F	Neg	Neg	RI equ	-	+	UE	RS
#10	62	F	Neg	Neg	Neg	Neg	+	FGE	RS
#11	51	M	LI equ	LI equ	RI equ	-	+	UE	RI
#12	51	F	Bilat. I	LI equ	Neg	-	+	UE	L

F: Female; M: Male; Neg: Negative; Bilat: Bilateral; L: Left; R: Right; I: Inferior; S: Superior; FGE: Four-gland exploration; U: Unilateral exploration; TT: Total thyroidectomy; equ: Equivocal
